# Preserving and utilizing microbial diversity for innovation and sustainability

**DOI:** 10.1099/mic.0.001544

**Published:** 2025-03-19

**Authors:** Nelson Lima

**Affiliations:** 1 MUM-Micoteca da Universidade do Minho, CEB-Biological Engineering Centre, University of Minho, Campus de Gualtar, 4710-057 Braga, Portugal; 2 LABBELS (Associate Laboratory, Braga/Guimarães), University of Minho, Campus de Gualtar, 4710-057 Braga, Portugal

**Keywords:** biobanks, culture collections, long-term preservation, microbial resources, networks, research infrastructures

## Abstract

Microbial culture collections have been fundamental to microbiology since their inception in the late nineteenth century. Initiated by Professor Král, the collections preserve and distribute microbial strains, enabling scientific advancements. Over time, they evolved into microbiological resource centres, integrating taxonomic expertise and adhering to international legal frameworks and quality management systems. Legal frameworks, including the Nagoya Protocol and biosecurity regulations, ensure ethical access and use of microbial resources. Regional networks, such as Microbial Resource Research Infrastructure – European Research Infrastructure Consortium at the European level, or, in the future, the Global Biological Resources Centres Network, coordinate efforts, fostering innovation and collaboration. Today, microbial culture collections support biotechnology, personalized medicine, agriculture and environmental sustainability. They also play a crucial role in public education, addressing misconceptions about microbes. As research progresses, these collections will continue to contribute to scientific discovery, bioeconomic growth and solutions to global challenges such as climate change, food security and ecosystem health.

## Culture collections

The first microbial culture collection dedicated to maintaining and supplying strains was established around 1890 by Professor Král at the German University of Prague, who published the first strain catalogue in 1900. This milestone was made possible by the pioneering work of British surgeon Joseph Lister in 1878 and German physician and microbiologist Robert Koch in 1881, both of whom first described pure culture isolation techniques. Additionally, French chemist Louis Pasteur, widely recognized as one of the founders of modern microbiology, played a crucial role in advancing the knowledge about microbes.

Following Král’s initiative, numerous other collections were established, some of which are still in operation. Over time, many more collections have been created to maintain a vast diversity of microbes, while others focus on specific taxonomic groups. Currently, using the information from the World Data Centre for Microorganisms (WDCM/CCINFO) [1], there are 866 culture collections registered from 80 countries and regions representing a total of 4,120,390 strains. Table 1 shows the distribution of the microbial culture collections and their holdings by continent.

**Table 1.** Distribution of culture collections and strains by continent, countries and regions

Continent	Countries and regions	Culture collections	Strains
Africa	9	27	21,548
America	11	217	624,970
Asia	21	318	2,111,432
Europe	34	273	1,227,602
Oceania	4	42	127,304

As a result, microbial culture collections have been fundamental to the advancement of microbial studies, evolving alongside the field of microbiology. Their primary functions include the long-term preservation, distribution and exchange of microbial strains worldwide, eliminating the need for repeated isolation whenever the scientific community requires strains from the same species.

## Culture collections as biological resource centres

**Fig. 1.**
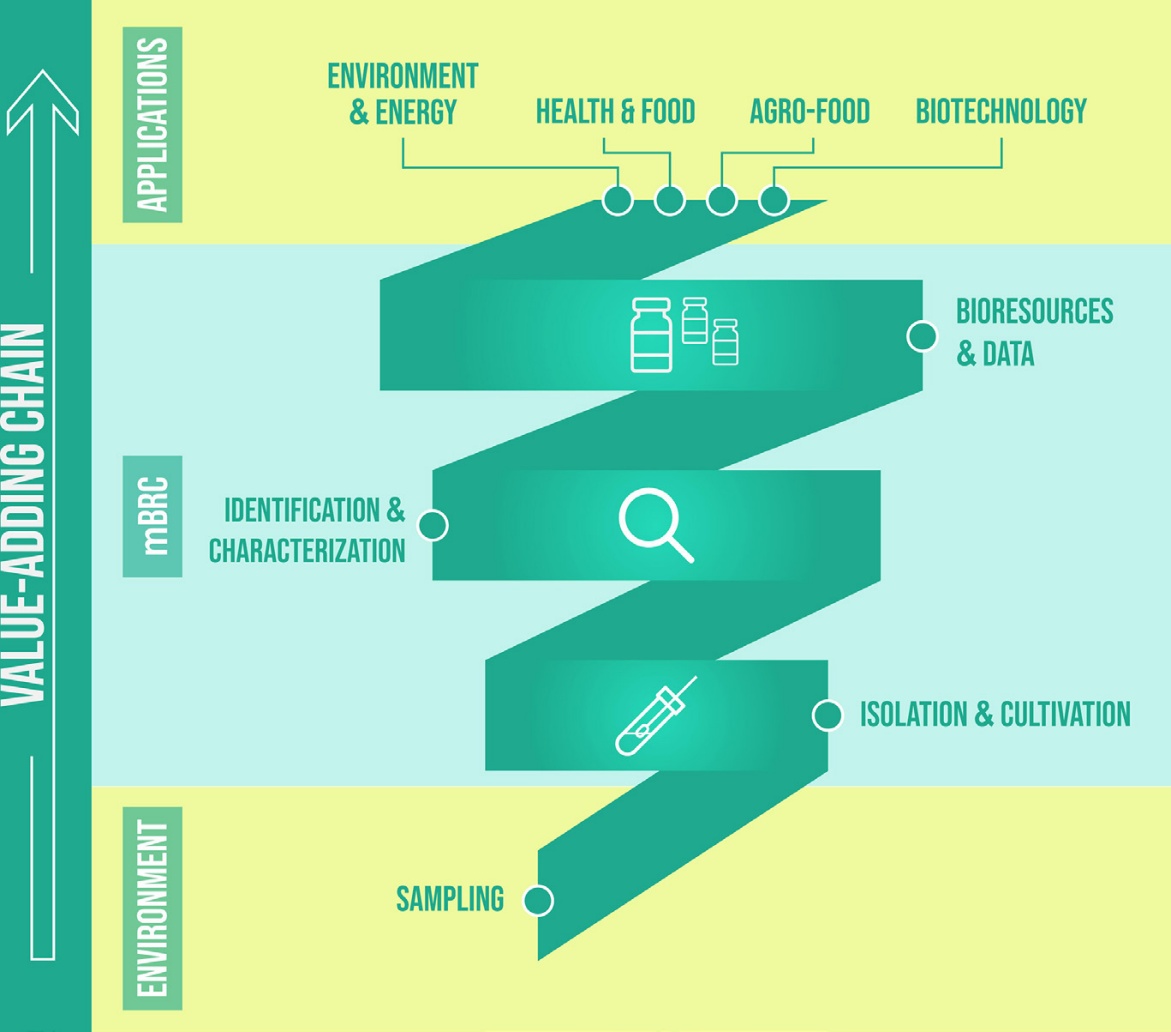
Value-adding chain in mBRCs from isolates to well-characterized strains preserved and supplied for different applications.

Microbial culture collections have evolved into more complex organizations, expanding the scope of their operations. Alongside each collection, taxonomic expertise has developed, along with other scientific competencies, to apply these resources to various applications. As a result, the range of services offered by culture collections has increased significantly, becoming a crucial part of their business (Fig. 1).

Additionally, with the advent of cutting-edge technology and the digital era, culture collections have adopted best practices, guidelines and legal frameworks to enhance their operations. This evolution culminated in the introduction of the concept of biological resource centres (BRCs) in 2001 by the Organisation for Economic Co-operation and Development (OECD) [2].

In essence, microbiological resource centres (mBRCs) form a vital component of the infrastructure supporting biotechnology. They serve as repositories and service providers, housing living cells, organism genomes and information related to heredity and biological system functions. mBRCs must adhere to the highest standards of quality and expertise required by the international scientific and industrial communities to ensure the reliable delivery of biological materials and information. With this new concept, the culture collections reinforced the implementation of quality management systems using the certification process, such as the ISO9001, or the accreditation of processes using the ISO 17025 standards.

Nowadays, the recent ISO 20387 standard specifically addresses the ‘General Requirements for Biobanking’, whereas biobanking is defined as the process of acquiring and storing, along with some or all of the activities related to collection, preparation, preservation, testing, analysing and distributing defined biological material as well as related information and data. In addition, this standard defines biological material as any substance derived or part obtained from an organic entity such as a human, animal, plant, micro-organism(s) or multicellular organism(s) that is (are) neither animal nor plant (e.g. brown seaweed and fungi).

Quality issues have become even more critical today as microbial culture collections recognize the need to collaborate within networks, whether at national, regional or international levels, often under shared legal research infrastructures. Establishing a robust quality framework is essential for defining their role at different levels, enhancing cooperation and improving communication with users. Standards applicable to mBRCs or biobanks can provide the necessary guidelines to ensure consistency, efficiency and responsiveness to user needs [3].

## Culture collection and the legal framework

The time that microbial culture collections, which are *ex situ* repositories of biological diversity, start to accomplish new regulatory and legal frameworks is mainly as a consequence of the adoption of the Convention of Biological Diversity at the Earth Summit in Rio de Janeiro on 5 June 1992 [4], which included access and benefit-sharing provisions and the Nagoya Protocol [5], which establishes a framework for the fair and equitable sharing of benefits arising from the utilization of genetic resources, ensuring that countries and communities providing these resources receive appropriate benefits [6].

Within the legal framework, the potential dual-use of micro-organisms has become more strictly regulated. This regulation ensures that microbes intended for research, industrial, agricultural or medical applications are not misused for unethical or harmful activities, such as bioterrorism or the development of biological weapons.

The Biological Weapons Convention [7] and national biosecurity regulations play a crucial role in governing the access, transfer and application of microbial resources. These frameworks require institutions and researchers to comply with biosafety, biosecurity and ethical guidelines, ensuring responsible handling, storage and distribution of microbes. Additionally, microbial culture collections and microbial biobanks need to put in place risk assessment procedures, traceability measures and user agreements to prevent unauthorized use while fostering legitimate use by the user communities.

## Culture collection organizations and networks

As mentioned above, the WDCM, in collaboration with the World Federation for Culture Collections, plays a global role in bringing together public service collections and delivering the Global Catalogue of Microorganisms and much more.

At the regional level, organizations such as the European Culture Collections’ Organisation, the Asian Consortium for the Conservation and Sustainable Use of Microbial Resources, the United States Culture Collections Network and the Latin American Federation for Culture Collections demonstrate how microbial culture collections cooperate across different regions.

More recently, the integration of the microbial domain with the human domain under the biobank concept has led to the establishment of the Lusophone Network of Biobanks and Biological Collections, further strengthening collaboration and resource sharing [8].

**Fig. 2.**
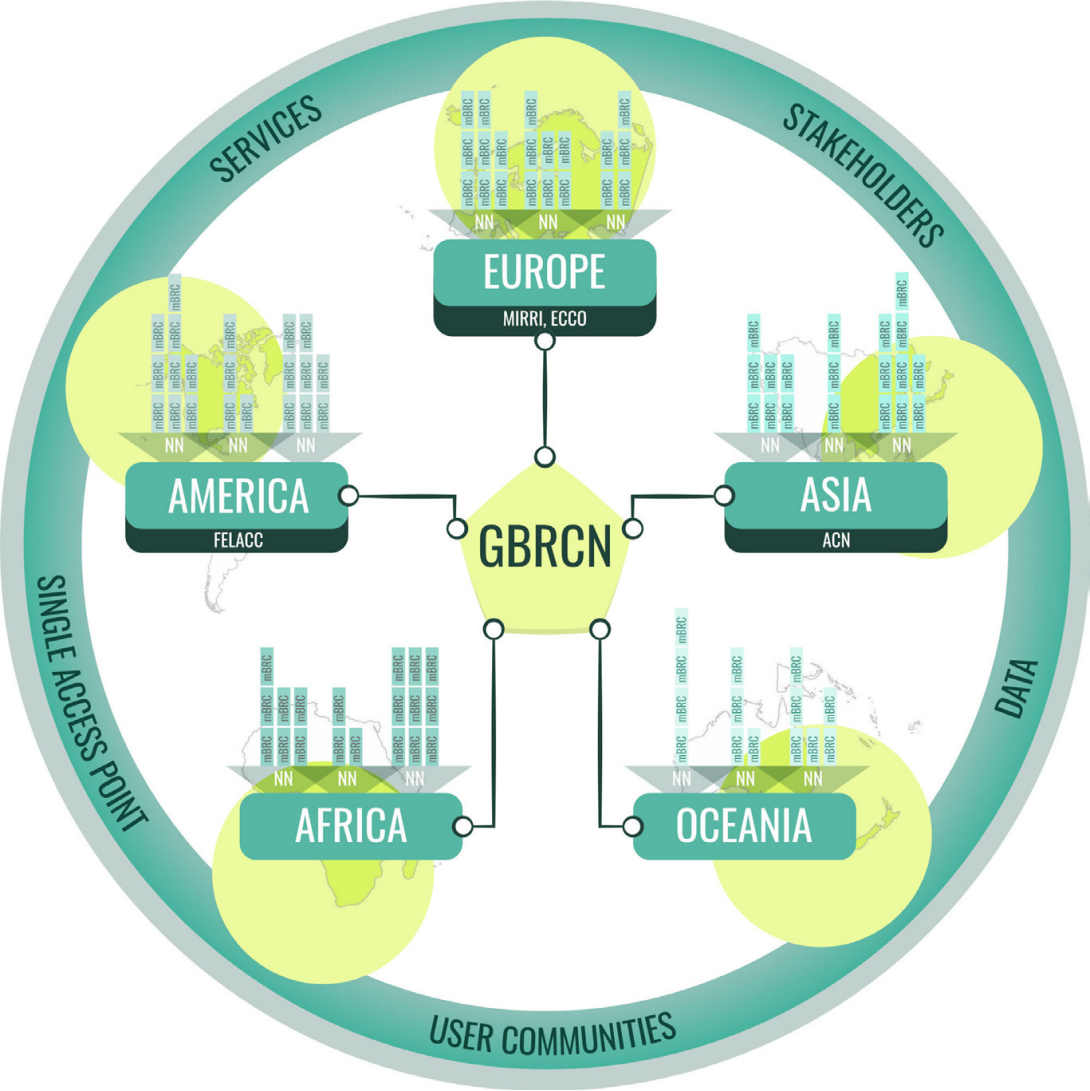
GBRCN is a single access point to global biological resources that integrates hierarchically regional networks built upon national NN of mBRCs. Unlike NN and mBRC, which focus on operational tasks, GBRCN also includes decision-making and executive bodies.

The OECD Task Force on BRC [2] envisioned integrating various national and regional networks to establish a distributed yet highly coordinated framework known as the Global Biological Resources Centres Network (GBRCN) [9]. This ‘network of networks’ aims to implement OECD best practices while reducing fragmentation and silos within mBRCs and their associated data. In doing so, it seeks to unlock its potential to drive economies of scale, minimize duplication among culture collections and enhance data interoperability. Finally, we can see the GBRCN federating different regional and national networks/nodes (NN) (Fig. 2).

A distinctive approach in Europe is Microbial Resource Research Infrastructure – European Research Infrastructure Consortium (MIRRI-ERIC), a legal entity that connects microbial culture collections and research institutions to provide high-quality microbial resources and services for research, industry and biotechnology.

MIRRI-ERIC operates under the European Strategy Forum on Research Infrastructures, promoting scientific excellence and the sustainable use of microbial biodiversity across Europe. It currently consists of seven-member and one-observer countries, each one with its National Node, and actively participates in or coordinates several European projects, including iSIDORE, CanServ, AgroServ, Bioindustry 4.0, Microbes, Microbes-4-Climate and MALDIbank.

As MIRRI-ERIC continues to expand, it plays a pivotal role in reorganizing European microbial culture collections, ensuring that they are prepared to address societal challenges while acting as a catalyst for innovation.

## Final remarks

In conclusion, microbial culture collections have been pivotal to science since the foundation of microbiology. They continuously contribute to the discovery and description of new species, expanding our understanding of microbial diversity and how we can preserve and utilize it. This is an immense task, considering that the total number of bacterial cells on Earth surpasses the estimated number of stars in the universe by several orders of magnitude. In addition, the human body hosts ten times more microbial cells than its own human cells.

The role of microbial culture collections in the interaction with lay citizens is vital as never before to avoid misconceptions about microbes and to support didactical activities in schools. In addition, they are actively involved in continuous training programmes, ensuring that intergenerational knowledge of how to handle microbes is preserved and passed on.

Over the years, they have also become key players in social and economic development [10,11]. In today’s knowledge-based society, microbial research has driven significant advancements and innovations in areas such as microbiomes, personalized medicine, precision and resilient agriculture, biopesticides and biofertilizers and clean water. Looking ahead, we hope this research will help mitigate climate change, secure food and feed supplies and promote a more sustainable future. Both ecosystems and humans depend on microbes, and fortunately, the majority of them are beneficial.

## Abbreviations

GBRCN: Global Biological Resources Centres Network
mBRCs: microbiological resource centres
MIRRI-ERIC: Microbial Resource Research Infrastructure – European Research Infrastructure Consortium
OECD: Organisation for Economic Co-operation and Development
WDCM: World Data Centre for Microorganisms
